# The Urine and Clinical Chemistry of the Gastric Contents, the Common Poisons, and Milk

**Published:** 1904-12

**Authors:** 


The Urine and Clinical Chemistry of the Gastric Contents, the Common Poisons, and Milk. By J. W. Holland, M.D., Professor of Medical Chemistry and Toxicology, Jefferson Medical College, Philadelphia. Seventh edition, revised and enlarged, with 41 illustrations. Philadelphia: P. Blakiston's Son & Company. 1904. (Price, $1.00 net.)

Here is a book which is remarkable in many ways in the outpouring of scientific works. One cannot help wondering sometimes in receiving circulars and prospectuses and announcements of "now ready," "in preparation," or "in press," if many of the writers of today are not running orthographic races with themselves. There seems to be cropping out in the medical literary world a sort of tendency to a riot of words, which when they are all digested or dissected and divested of all outward pomp and glory mean little else than the self-glorification of a few isolated ideas.

In Holland's work on the urine and the clinical chemistry of the gastric contents, the common poisons and milk, there is an entire absence of all preliminary. It is all meat; all good, solid sensible, workable information. There is a title page and a table of contents, but no preface, no foreword, no introduction, for which much thanks. Considerable space is thereby made available for much valuable material. If there is any physician who knows all about the urine in health and disease, and who can draw his own deductions from an urinalysis, he doesn't need this book. But for the everyday man it is the best work of its kind which has been issued between covers in a long time.

The book is handy, it is complete, and the working plan, as laid down, can hardly be improved upon. It explains the whys and wherefores in simple, understandable language, and it makes comparatively easy the boggy road of urine analysis upon which so many well-meaning but misguided workers have found themselves floundering amid doubt and dubious diagnoses.

The information in other departments is just as clearly set forth as that devoted to the urine. The book is freely illustrated, well-printed and has been markedly improved since the first of the six previous editions was published. N. W. W.

